# Data on secondary structures and ligand interactions of G-rich oligonucleotides that defy the classical formula for G4 motifs

**DOI:** 10.1016/j.dib.2017.02.023

**Published:** 2017-02-12

**Authors:** Maria Vlasenok, Anna Varizhuk, Dmitry Kaluzhny, Igor Smirnov, Galina Pozmogova

**Affiliations:** a Research and Clinical Center for Physical Chemical Medicine, 119435 Moscow, Russia; b Moscow Institute of Physics and Technology (State University), 117303 Moscow, Russia; c Engenlhardt Institute of Molecular Biology, 119991 Moscow, Russia

**Keywords:** G-quadruplexes, G4 motifs, Thermal stability, G4 ligands

## Abstract

The data provided in this article are related to the research article "The expanding repertoire of G4 DNA structures" [1]. Secondary structures of G-rich oligonucleotides (ONs) that represent “imperfect” G-quadruplex (G4) motifs, i.e., contain truncated or interrupted G-runs, were analyzed by optical methods. Presented data on ON structures include circular dichroism (CD) spectra, thermal difference spectra (TDS) and UV -melting curves of the ONs; and rotational relaxation times (RRT) of ethidium bromide (EtBr) complexes with the ONs. TDS, CD spectra and UV-melting curves can be used to characterize the topologies and thermal stabilities of the ON structures. RRTs are roughly proportional to the hydrodynamic volumes of the complexes and thus can be used to distinguish between inter- and intramolecular ON structures. Presented data on ON interactions with small molecules include fluorescence emission spectra of the G4 sensor thioflavin T (ThT) in complexes with the ONs, and CD-melting curves of the ONs in the presence of G4-stabilizing ligands N-methylmesoporphyrin IX (NMM) and pyridostatin (PDS). These data should be useful for comparative analyses of classical G4s and “defective”G4s, such as quadruplexes with vacancies or bulges.

**Specifications Table**

**Table:** 

Subject area	*Molecular biology; Physical chemistry*
More specific subject area	*DNA secondary structures*
Type of data	*Table, Figures*
*How data was acquired*	*MALDI TOF MS (Microflex mass spectrometer, Bruker); UV, CD and fluorescence spectroscopy (Chirascan spectrophotometer, Applied Photophysics); fluorescence polarization measurements (Cary Eclipse fluorescence spectrophotometer, Agilent Technologies); fluorescence decay measurements (Easy Life V fluorescence lifetime fluorometer, Optical Building Blocks Corporation)*
*Data format*	*Raw, Analyzed*
*Experimental factors*	*ON solutions in the specified buffers were denatured at 95* *°C for 5* *min and snap cooled on ice prior to the experiments.*
*Experimental features*	*UV absorbance, CD and fluorescence emission spectra, and UV-melting curves were recorded on a spectrophotometer equipped with thermoregulated cuvette holder. Fluorescence rotational relaxation times were calculated using fluorescence polarization and fluorescence lifetime values.*
*Data source location*	*Research and Clinical Center for Physical Chemical Medicine and Engelhardt Institute of Molecular Biology, Moscow, Russian Federation*
*Data accessibility*	*The data is available within this article*

**Value of the data**

• Data allow for assessment of relative thermodynamic stabilities of imperfect G4 structures with single defects and classical (perfect) G4s.
• Data allow one to evaluate sensitivities of G4s with defects towards known G4-stabilizing ligands (NMM and PDS) and a popular ligh-up probe (ThT).
• Data can be compared with the previously published data on G4s with vacancies or bulges and used for developing improved G4-predicting algorithms.

## 1 Data

**Fig. 1:**
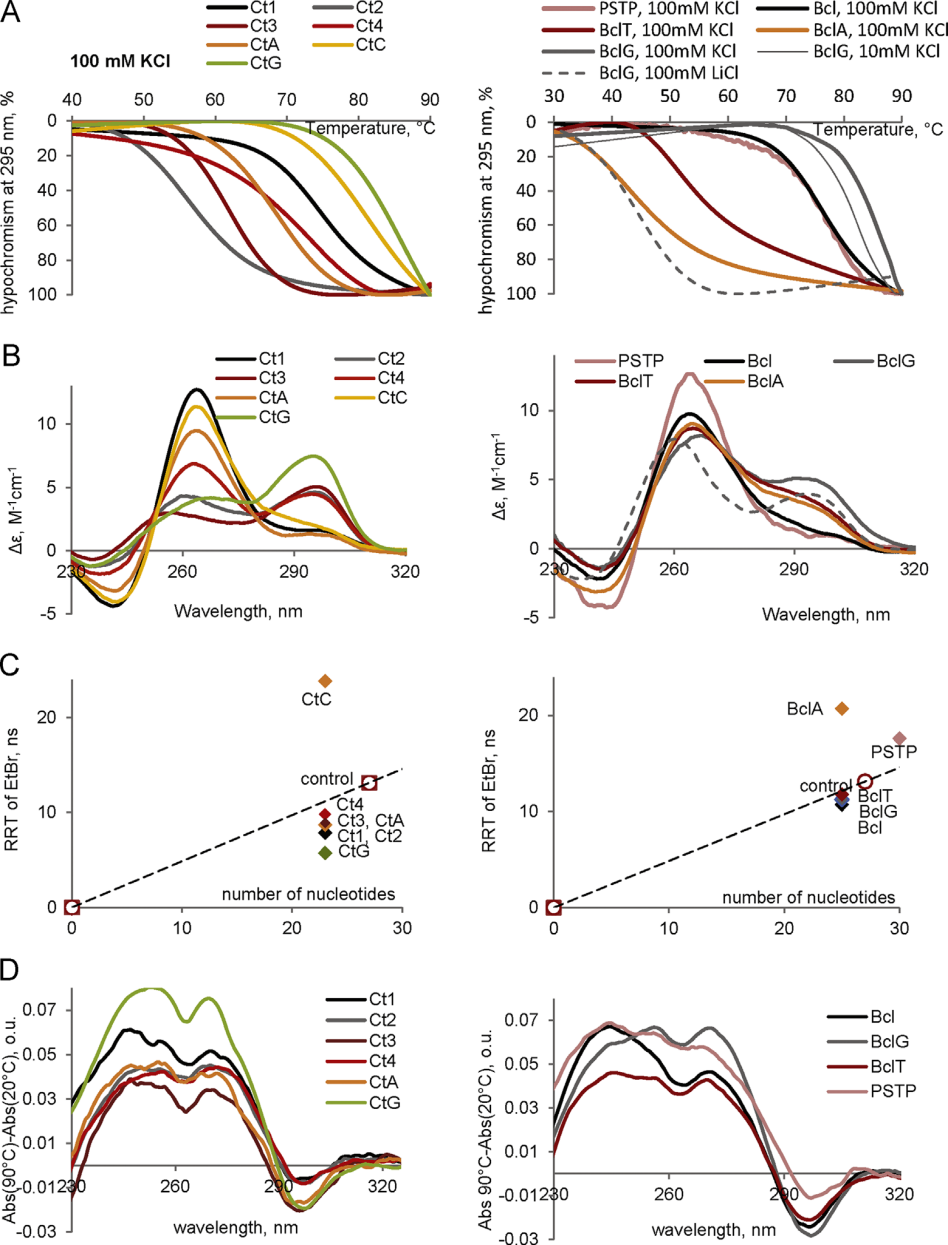
Characterization of genomic imGQ ONs and their derivatives by optical methods. A: UV-melting curves. B: CD spectra. The ellipticity is given per mole of nucleotide. Buffer conditions: 25 mM Tris–HCl (pH 7.5) and 100 mM КCl (unless otherwise specified; solid lines) or 100 mM LiCl (dotted lines). ON concentration was 1.5 μM. С: Rotational relaxation times (RRT) of EtBr in complexes with the ONs. The RRT values were calculated based on the measured values of fluorescence polarization and fluorescence lifetime. Control is a 27-mer hairpin. Temperature: 20 °C. ON concentration was 5 μM. D: TDS spectra. Buffer conditions: 25 mM Tris–HCl (pH 7.5) and 10 mM КCl. ON concentration was 1.5 μM.

**Fig. 2:**
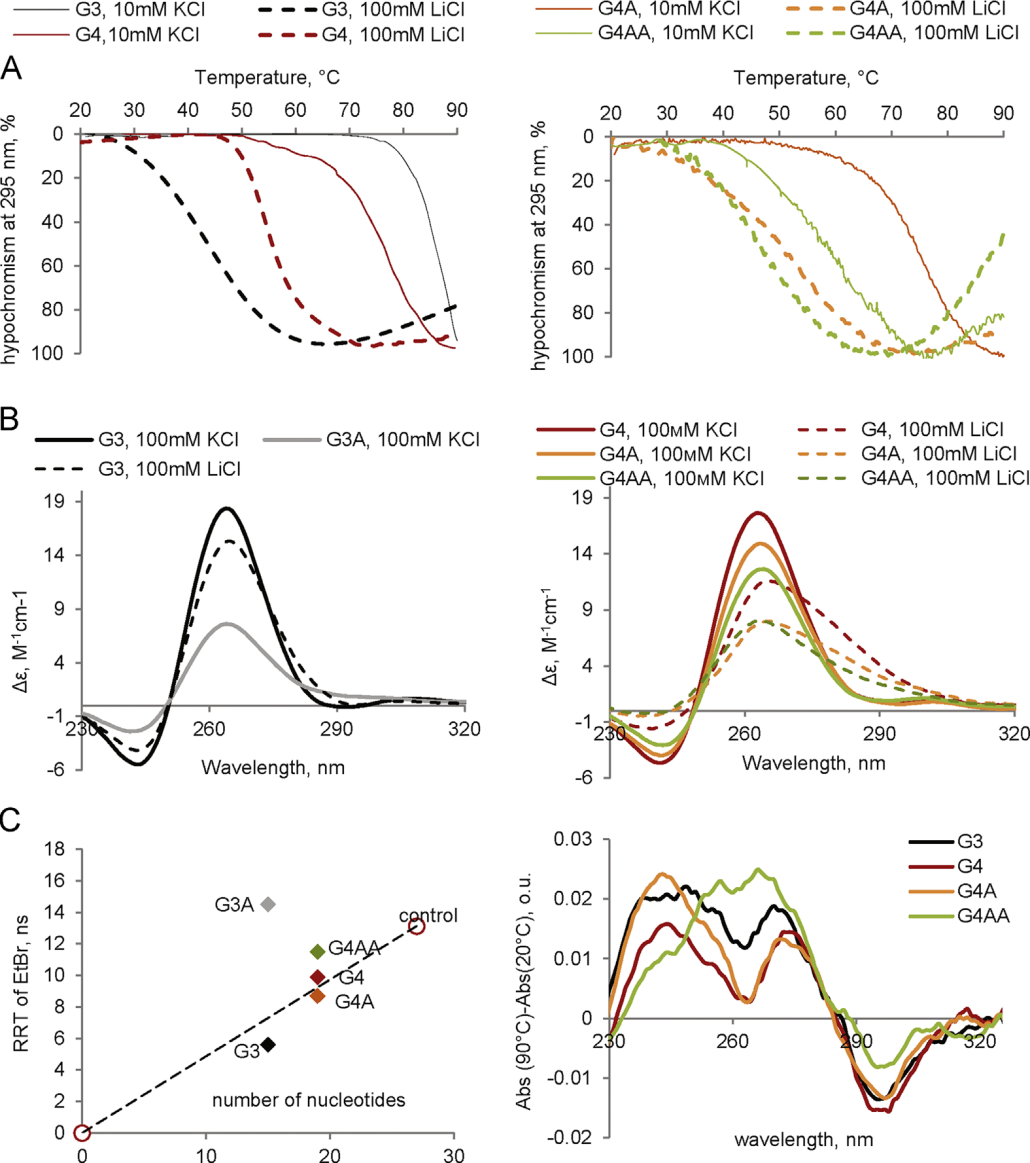
Characterization of model GQ and imGQ ONs by optical methods. A: UV-melting curves. B: CD spectra. The ellipticity is given per mole of nucleotide. Buffer conditions: 25 mM Tris–HCl (pH 7.5). КСl or LiCl concentrations are specified in the figure legends. ON concentration was 1.5 μM. С: Rotational relaxation times (RRT) of EtBr in complexes with the ONs. Temperature: 20 °C. ON concentration was 5 μM. D: TDS spectra. Buffer conditions: 25 mM Tris–HCl (pH 7.5) and 10 mM КCl. ON concentration was 1.5 μM.

**Fig. 3:**
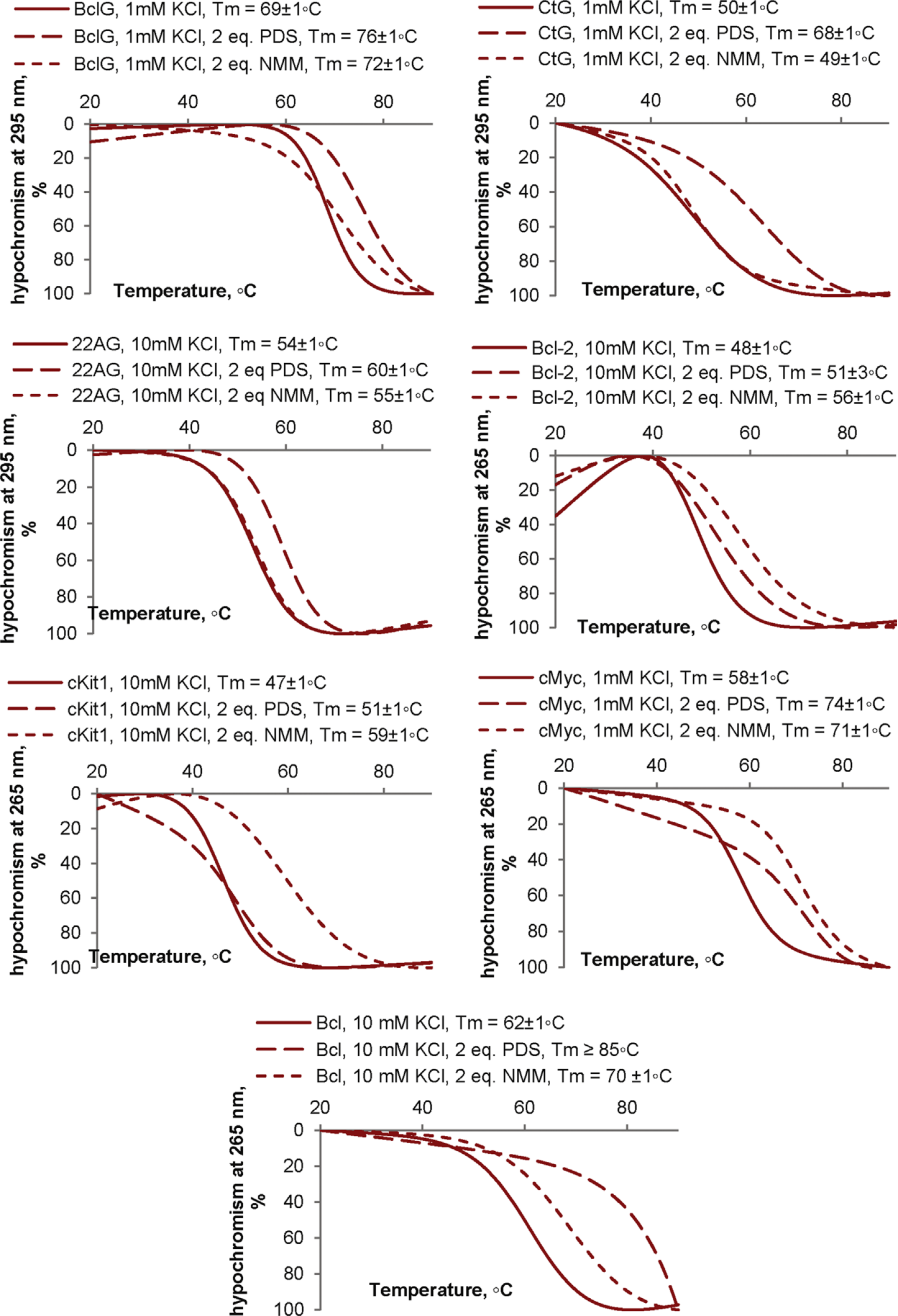
GQ stabilization by N-methylmesoporphyrin IX (NMM) and piridostatin (PDS). Melting by CD. Conditions: 20 mM Tris–HCl, 1.5 μM ON, 3 μM PDS/NMM. KCl concentrations are specified in the figure legends.

**Fig. 4:**
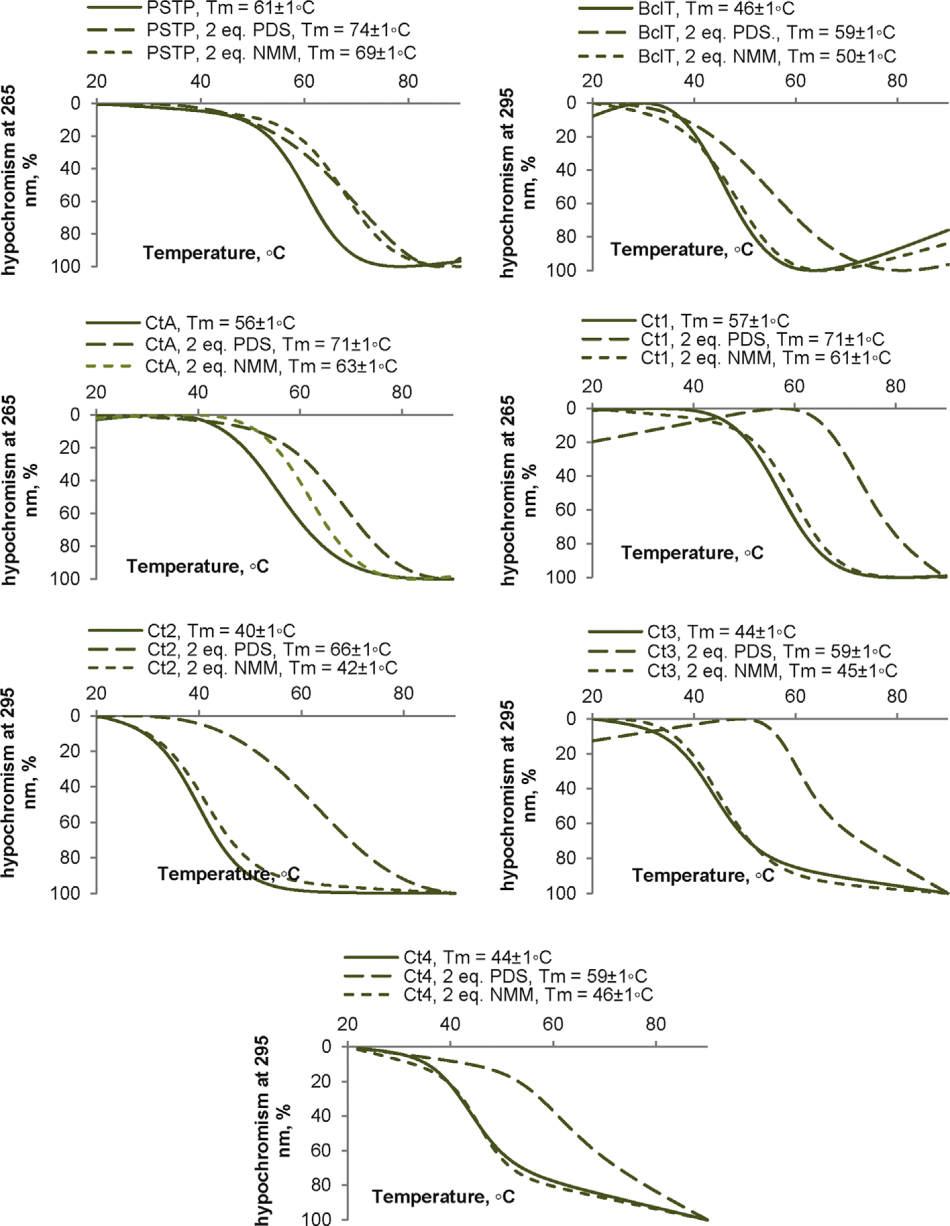
imGQ stabilization by N-methylmesoporhhyrin IX (NMM) and piridostatin (PDS). Melting by CD. Conditions: 20 mM Tris–HCl, 10 mM KCl, 1.5 uM ON, 3 μM PDS/NMM.

**Fig. 5:**
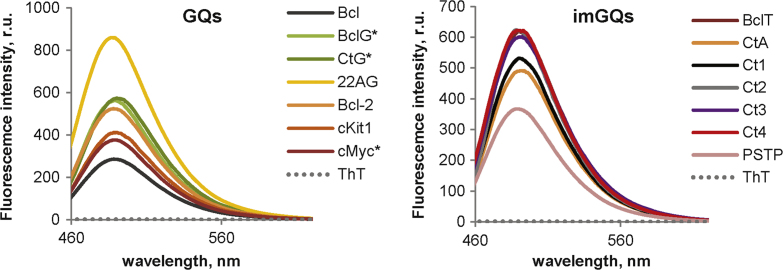
GQ and imGQ interactions with thioflavin T (ThT). Conditions: 20 mM Tris–HCl, 10 mM KCl (* = 1 mM KCl), 1.5 μM ON, 1.5 μM ThT. Excitation at 425 nm. BclT and Ct2 spectra almost coinside with the Ct2 spectrum.

The dataset of this article provides information on G-rich ONs that defy the consensus G_3+_N_L1_G_3+_N_L2_G_3+_N_L3_G_3+_ formula for G4 motifs and may form “imperfect” quadruplex structures (imGQs) with bulges between G-tetrads or vacancies/mismatches in the tetrads [2], [3]. Table 1 contains MS data on imGQ ONs and control (GQ) ONs. GQ ONs are “perfect” G4s that comply with the G_3+_N_L1_G_3+_N_L2_G_3+_N_L3_G_3+_ formula. The ON set includes both genomic and model sequences. Fig. 1 contains data on secondary structures of genomic ONs and their mutants. Fig. 2 contains data on model ONs. Fig. 3, Fig. 4 show NMM [4] and PDS [5] effects on thermal stabilities of GQs and imGQs, respectively. Fig. 5 shows ThT [6] fluorescence in complexes with GQs and imGQs.

## 2 Experimental design, materials and methods

Sequences of ONs CT1, PSTP and Bcl were chosen randomly from G-rich fragments of the human genome, chromosome 18 (chr18: +46379322 to +46379344, chr18: −43572049 to −43572072, chr18: −60985942 to −60985966, respectively, NCBI Reference Sequence: NC_000018.9). ONs Ct2-Ct4, CtA, CtC and CtG are Ct1 mutants. BclT, BclA and BclG are Bcl mutants. G3, G3A, G4, G4A and G4AA are model ONs (putative perfect and imperfect G4s with short loops). 22AG, Bcl-2, cKit1 and cMyc are control well-characterized G4s (see [7] and references therein).

ON synthesis, HPLC purification and MALDI TOF MS analysis (Table 1) were performed as previously described [8]. Absorption, CD, and fluorescence emission spectra were recorded using a Chirascan spectrophotometer (Applied Photophysics, UK). Molar CD per nucleotide residue (Figs. 1A and 2A) was calculated as follows: Δε=θ/(32.982×C×l×n), where θ is ellipticity (degree), C is ON concentration (M); l is optical pathlength (cm) and n is the number of nucleotide residues in the ON. In the melting experiments (Figs. 1B and 2B), ON absorbance at 295 nm was registered every 1 °C across the 20–90 °C temperature range (see Table 2 in [1] for Tm values). The heating rate was 1 °C /min. Rotational relaxation times (RRT) of ethidium bromide (EtBr) complexes with the ONs (Figs. 1C and 2C) were estimated using the PerrineWeber equation [9], [10]: RRT=3τ(1/P_0_−1/3)/(1/P−1/P_0_), where P is observed polarization, P_0_=41±1% is its limiting value in the absence of rotational depolarization, and τ is fluorescence lifetime of EtBr in complexes with the ONs. The fluorescence polarization P was calculated as previously described [9]: P=(I_||_−I_┴_)/(I_||_+I_┴_). The vertical (I_||_) and horizontal (I_┴_) components of EtBr fluorescence intensity at emission maximum (610 nm) were measured with Cary Eclipse spectrofluorometer at 4 °C upon excitation at 540 nm by the vertically polarized light. Concentration of EtBr was 1 μM, and ON concentration was 5 μM. The fluorescence lifetime (τ) was evaluated using Easy Life V. Fluorescence decay was registered through a RG610 long pass filter at excitation LED 525 nm. Thermal difference spectra (TDS, Figs. 1D and 2D) were obtained by subtracting ON absorption spectra recorded at 20 °C from the spectra recorded at 90 °C.

In the melting experiments with NMM and PDS (Fig. 3, Fig. 4), the ligands were added to pre-annealed ON solutions, and ON CD at 265/295 nm was registered every 1 °C across the 20–90 °C temperature range. The heating rate was 1 °C /min. The melting temperatures of GQs/imGQs were defined by performing a fitting procedure using the two-state model for monomolecular melting [11] in DataFit 9. PDS was obtained from ApexBio, and NMM was obtained from Frontier Scientific. Fluorescence emission spectra of ThT in complexes with the ONs (Fig. 5) were recorded using a Chirascan spectrophotometer (Applied Photophysics) upon excitation at 425 nm at 20 °C. ThT was obtained from Abcam.

## Figures and Tables

**Table 1:** Sequences of oligodeoxyribоnucleotides (ONs) and MALDI-TOF MS data. Interrupted or truncated G_3+_ runs are in Italics and bold.

ON code	Sequence, 5′-3′	m/z [M + H]+ found (calculated.)
Ct1	***GGTG***ACAGGGGTATGGGGAGGGG	7335 (7332)
Ct2	GGGGACA***GGTG***TATGGGGAGGGG	7332 (7332)
Ct3	GGGGACAGGGGTAT***GGTG***AGGGG	7333 (7332)
Ct4	GGGGACAGGGGTATGGGGA***GGTG***	7333 (7332)
CtG	GGGGACAGGGGTATGGGGAGGGG	7358 (7357)
CtA	***GGAG***ACAGGGGTATGGGGAGGGG	7340 (7340)
CtC	***GGCG***ACAGGGGTATGGGGAGGGG	7319 (7317)
PSTP	***GGTG***AATGGGGCAGTGGGGTGGGG	7651 (7652)
Bcl	GGGGGCCGTGGGGTGGGAGCTGGGG	7950 (7958)
BclG	GGGGGCCGTGGGGTGGGGGCTGGGG	7978 (7974)
BclA	GGGGGCCGTGGGGT***GAGAG***CTGGGG	7946 (7942)
BclT	GGGGGCCGTGGGGT***GTGAG***CTGGGG	7933 (7933)
G3	GGGTGGGTGGGTGGG	4805 (4802)
G3A	GGGT***GAG***TGGGTGGG	4786 (4786)
G4	GGGGTGGGGTGGGGTGGGG	6118 (6119)
G4A	GGGGT***GAGG***TGGGGTGGGG	6104 (6103)
G4AA	GGGGT***GAGA***TGGGGTGGGG	6088 (6087)
22AG	AGGGTTAGGGTTAGGGTTAGGG	6967 (6968)
Bcl-2	GGGCGCGGGAGGGAATTGGCGGGG	7624 (7622)
cKit1	GGGAGGGCGCTGGGAGGAGGG	6699 (6699)
cMyc	TGAGGGTGGGTAGGGTGGGTAA	6994 (6993)
